# Impact of aging on the clinical outcomes of Japanese patients with coronary artery disease after percutaneous coronary intervention

**DOI:** 10.1007/s00380-013-0339-9

**Published:** 2013-04-04

**Authors:** Hidehiro Kaneko, Junji Yajima, Yuji Oikawa, Shingo Tanaka, Daisuke Fukamachi, Shinya Suzuki, Koichi Sagara, Takayuki Otsuka, Shunsuke Matsuno, Ryuichi Funada, Hiroto Kano, Tokuhisa Uejima, Akira Koike, Kazuyuki Nagashima, Hajime Kirigaya, Hitoshi Sawada, Tadanori Aizawa, Takeshi Yamashita

**Affiliations:** Department of Cardiovascular Medicine, The Cardiovascular Institute, 3-2-19, Nishiazabu, Minato-ku, Tokyo, 106-0031 Japan

**Keywords:** Aging, Japanese, Coronary artery disease, Prognosis

## Abstract

Japan has become an aging society, resulting in an increased prevalence of coronary artery disease. However, clinical outcomes of elderly Japanese patients after percutaneous coronary intervention (PCI) remain unclear. Of the 15,227 patients in the Shinken Database, a single-hospital-based cohort of new patients, 1,214 patients who underwent PCI, was evaluated to determine the differences in clinical outcomes between the elderly (≥75 years) (*n* = 260) and the non-elderly (<75 years) (*n* = 954) patients. A major adverse cardiac event (MACE) was defined as a composite end point, including all-cause death, myocardial infarction (MI), and target lesion revascularization. Male gender and obesity were less common, and the estimated glomerular filtration rate (eGFR) was significantly lower in the elderly than in the non-elderly. Left ventricular ejection fraction (LVEF) was comparable between these groups. Left main trunk disease and multivessel disease were more common in the elderly than in the non-elderly group. Occurrence of MACE was frequent, and the incidences of all-cause death, cardiac death, and the admission rate for heart failure were significantly higher in the elderly patients. Multivariate analysis showed that prior MI, low eGFR, and poor LVEF were independent predictors for all-cause death in the elderly patients. Elderly patients had worse clinical outcomes than the non-elderly patients. Low eGFR and LVEF were independent predictors of all-cause death after PCI, suggesting that left ventricular dysfunction and renal dysfunction might synergistically contribute to the adverse clinical outcomes of the elderly patients undergoing PCI.

**Electronic supplementary material:**

The online version of this article (doi:10.1007/s00380-013-0339-9) contains supplementary material, which is available to authorized users.

## Introduction

Japan has the highest proportion of elderly citizens in the world. In 1989, only 11.6 % of the population was aged ≥65 years. However, the Ministry of Internal Affairs and Communications recently reported that 23.8 % of the population is aged ≥65 years, and 11.8 % of the population is ≥75 years. In parallel with the aging population in Japan, coronary artery disease (CAD) has become a common degenerative condition. For the management of CAD, elucidating the effect of aging on the clinical characteristics and outcomes of Japanese CAD patients, as well as clarifying the prognostic factors associated with CAD, is important.

The TIME trial showed that patients aged 75 years or older with angina refractory to standard drug therapy benefited more from revascularization than from optimized medical therapy in terms of angina relief and improvement in quality of life; this finding supports the clinical efficacy of revascularization therapy in elderly patients [1]. Because of wide variations in ethnicity, geographic locations, social health care systems, and treatment strategies [2–8], it has been suggested that the clinical outcomes of CAD in Japanese patients are likely to be different from those in the Western population. Furthermore, percutaneous coronary intervention (PCI) is preferred and widely performed in CAD patients in Japan.

Clinical studies focusing on elderly Japanese patients undergoing PCI are limited. The prognosis of elderly Japanese CAD patients treated using PCI remains to be determined. We conducted a hospital-based cohort study using the Shinken Database 2004–2010 to investigate the prevalence and prognosis of patients with cardiovascular diseases in Japan [9]. The present study, which included patients who underwent PCI (*n* = 1,214), aimed to identify the clinical characteristics and outcomes in elderly CAD patients after PCI in a Japanese urban city.

## Patients and methods

### Study patients and protocols

The Shinken Database included all new patients visiting the Cardiovascular Institute in Tokyo, Japan (“Shinken” is an abbreviated name for the hospital in Japanese), and excluded cancer patients and any foreign travelers. This hospital-based database was established for the surveillance of the prevalence and prognosis of cardiovascular diseases in urban areas in Japan [10]. The registry was started in June 2004, and thereafter patients have been continually registered to the database annually. Data entered into the database between June 2004 and March 2011 (Shinken Database 2004–2010), which included 15,227 new patients, were used in the present study. Of these patients, only those who underwent PCI (*n* = 1214) were enrolled in the study. We obtained the following data: age; gender; height; body weight; history of prior myocardial infarction (MI), PCI, and coronary artery bypass graft (CABG); coronary risk factors; laboratory data; and medications at primary PCI. Ultrasound cardiography was routinely performed before PCI.

### Patient follow-up

The health status details of patients and the incidence of cardiovascular events and mortality were maintained in the database, and could be accessed through a link to the medical records of the hospital and through survey documents sent once a year to those who stopped hospital visits or were referred to other hospitals.

In the present analysis, the follow-up data recorded after April 1, 2011, were excluded. Therefore, the end of the follow-up period was defined by one of the following three criteria: (1) death before March 31, 2011; (2) the date of final hospital visit or response to our survey documents on prognosis, with a confirmation of the patient being alive on March 31, 2011; and (3) March 31, 2011, if the dates of death, final hospital visit, or final response to survey documents on prognosis were later than April 1, 2011.

### Ethics

The ethical committee at the Cardiovascular Institute authorized this study, and all patients gave written informed consent.

### Definitions

We defined elderly patients (≥75 years), whereas non-elderly patients (≤75 years). The death of patients was confirmed using the medical records of our hospital or via the information obtained from follow-up visits. Body mass index (BMI) was calculated at initial PCI by dividing the patient’s measured weight (kg) by the square of the height (m), and obesity was defined as a BMI of ≥25 kg/m^2^. The estimated glomerular filtration rate (eGFR) was calculated using the following GFR equation: GFR = 194 × (serum creatinine) − 1.094 × (age) − 0.287 × (0.739, if female) [11]. Target lesion revascularization (TLR) was defined as any repeat revascularization procedure (percutaneous or surgical) at the original target lesion site, which included the stented area plus a margin (typically 5 mm proximal and distal to the stent). A major adverse cardiac event (MACE) was defined as a composite end point of all-cause death, MI, and TLR. Cardiovascular death included death resulting from acute myocardial infarction, sudden cardiac death, death due to heart failure, death due to stroke, and death due to other cardiovascular causes.

### Statistical analysis

The categorical and continuous data of patients are presented as number (%) and mean ± standard deviation, respectively. The unpaired *t* test was used for comparisons of continuous variables between the two groups. Chi-square analysis was used to compare categorical variables. Long-term event-free survival was estimated using the Kaplan–Meier curves, and the log-rank test was used to assess the significance of differences between elderly and non-elderly patients. Univariate Cox regression analysis was used to identify the cofactors with significant effects on all-cause death and cardiovascular death. Multivariate Cox regression analysis was performed to determine the independent prognostic factors for all-cause death and cardiovascular death. *P* < 0.05 was considered to indicate statistical significance. These analyses were performed using SPSS version 19.0 software (SPSS, Chicago, IL, USA).

## Results

### Patients’ characteristics

Of the 1214 patients, 260 (21 %) were elderly (≥75 years) and 954 (79 %) were non-elderly. The median follow-up period was 1032 ± 704 days. Mean ages were 80.0 ± 4.3 years in the elderly patients and 61.4 ± 8.4 years in the non-elderly patients. Prevalence of stable angina pectoris and acute coronary syndrome (ACS) (non-ST elevation ACS and ST-elevation myocardial infarction) were comparable between the elderly and the non-elderly patients. Male gender (64.6 vs 87.3 %, *P* < 0.001), dyslipidemia (51.2 vs 61.8 %, *P* = 0.002), obesity (22.2 vs 43.8 %, *P* < 0.001), cigarette smoking (16.2 vs 42.5 %, *P* < 0.001), and family history of CAD (11.9 vs 17.0 %, *P* = 0.048) were less common in the elderly patients than in the non-elderly patients. Levels of total cholesterol (184.8 ± 35.3 vs 194.4 ± 39.6 mg/dl, *P* = 0.001), low-density lipoprotein (LDL) cholesterol (108.7 ± 30.5 vs 114.4 ± 33.9 mg/dl, *P* = 0.010), triglycerides (TG) (111.4 ± 53.2 vs 162.0 ± 118.8 mg/dl, *P* < 0.001), and hemoglobin A1c (HbA1c) (6.0 ± 1.1 % vs 6.2 ± 1.3 %, *P* = 0.034) were significantly lower in the elderly than in the non-elderly patients. The high-density lipoprotein (HDL) cholesterol level (54.2 ± 14.4 vs 50.0 ± 14.8 mg/dl, *P* < 0.001) was significantly higher in the elderly than in the non-elderly patients.

The use of dual antiplatelet therapy (93.1 vs 96.0 %, *P* = 0.045) and statins (50.4 vs 63.4 %, *P* < 0.001) was less common in the elderly than in the non-elderly patients. The use of vasodilators (46.2 vs 32.9 %, *P* < 0.001), diuretics (21.2 vs 10.3 %, *P* < 0.001), and aldosterone antagonists (9.2 vs 4.7 %, *P* = 0.005) was more common in the elderly than in the non-elderly patients. The prevalence of bare-metal stents and drug-eluting stents was comparable between the elderly and the non-elderly patients (Table 1).

**Table 1:** Patients’ background characteristics

	Non-elderly (*n* = 954)	Elderly (*n* = 260)	*P* value
Age (years)	61.4 ± 8.4	80.0 ± 4.3	<0.001
Male sex	833/954 (87.3)	168/260 (64.6)	<0.001
SAP	595/954 (62.4)	152/260 (58.5)	0.251
ACS	359/954 (37.6)	108/260 (41.5)	0.251
Non-STE ACS	186/954 (19.5)	52/260 (20.0)	
STEMI	173/954 (18.1)	56/260 (21.5)	
Prior MI	107/954 (11.2)	28/260 (10.8)	0.839
Prior PCI	100/954 (10.5)	29/260 (11.2)	0.755
Prior CABG	34/954 (3.6)	13/260 (5.0)	0.287
Hypertension	615/954 (64.5)	164/260 (63.1)	0.679
Diabetes mellitus	332/954 (34.8)	79/260 (30.4)	0.182
Dyslipidemia	590/954 (61.8)	133/260 (51.2)	0.002
Hyperuricemia	237/954 (24.8)	67/260 (25.8)	0.760
Obesity	417/953 (43.8)	56/252 (22.2)	<0.001
Cigarette smoking	405/954 (42.5)	42/260 (16.2)	<0.001
Family history	162/954 (17.0)	31/260 (11.9)	0.048
eGFR (ml/min/1.73 m^2^)	70.1 ± 21.4	57.3 ± 18.8	<0.001
Total cholesterol (mg/dl)	194.4 ± 39.6	184.8 ± 35.3	0.001
LDL cholesterol (mg/dl)	114.4 ± 33.9	108.7 ± 30.4	0.010
HDL cholesterol (mg/dl)	50.0 ± 14.8	54.2 ± 14.4	<0.001
TG (mg/dl)	162.0 ± 118.9	111.4 ± 53.2	<0.001
Glucose (mg/dl)	134.0 ± 55.8	135.5 ± 53.3	0.709
HbA1c (%)	6.2 ± 1.3	6.0 ± 1.1	0.034
LVEF (%)	61.6 ± 12.6	61.6 ± 14.3	0.985
DAPT	916/954 (96.0)	242/260 (93.1)	0.045
Anticoagulant therapy	67/954 (7.0)	26/260 (10.0)	0.110
Statins	605/954 (63.4)	131/260 (50.4)	<0.001
β-Blockers	354/954 (37.1)	94/260 (36.2)	0.778
ACE-Is	155/954 (16.2)	41/260 (15.8)	0.853
ARBs	363/954 (38.1)	107/260 (41.2)	0.362
RAS-Is	506/954 (53.0)	145/260 (55.8)	0.434
CCBs	426/954 (44.7)	113/260 (43.5)	0.732
Vasodilators	314/954 (32.9)	120/260 (46.2)	<0.001
Diuretics	98/954 (10.3)	55/260 (21.2)	<0.001
Aldosterone antagonists	45/954 (4.7)	24/260 (9.2)	0.005
Antidiabetic drugs	189/954 (19.8)	49/260 (18.8)	0.728
Thiazolidinediones	33/954 (3.5)	7/260 (2.7)	0.539
Insulin	33/954 (3.5)	5/260 (1.9)	0.207
LMT	45/954 (4.7)	27/260 (10.4)	0.001
MVD	558/954 (58.5)	171/260 (65.8)	0.034
BMS	303/954 (31.8)	90/260 (34.6)	0.383
DES	635/954 (66.6)	161/260 (61.9)	0.163

Data are expressed as mean ± standard deviation, or counts (percentage)

*SAP* stable angina pectoris, *ACS* acute coronary syndrome, *Non-STE ACS* non-ST-elevation ACS, *STEMI* ST-elevation myocardial infarction, *Prior MI* prior history of myocardial infarction, *Prior PCI* prior history of percutaneous coronary intervention, *Prior CABG* prior history of coronary artery bypass graft, *eGFR* estimated glomerular filtration rate, *LDL* low-density lipoprotein, *HDL* high-density lipoprotein, *TG* triglyceride, *LVEF* left ventricular ejection fraction, *DAPT* dual antiplatelet therapy, *Statin* 3-hydroxy-3-methylglutaryl coenzyme A (HMG-CoA) inhibitor, *ACE-I* angiotensin-converting enzyme inhibitor, *ARB* angiotensin II receptor blocker, *RAS-I* renin–angiotensin system inhibitor, *CCB* calcium-channel blocker, *LMT* left main trunk disease, *MVD* multivessel disease, *BMS* bare-metal stent, *DES* drug-eluting stent

### Echocardiography findings

Ultrasound cardiography showed that the left ventricular ejection fraction (LVEF) (62 ± 14 vs 62 ± 13 %, *P* = 0.985) was comparable between the elderly and non-elderly patients (Table 1).

### Angiographic findings

Coronary angiography showed that the number of patients with left main trunk (LMT) disease (10.4 vs 4.7 %, *P* = 0.001) and multivessel disease (MVD) (65.8 vs 58.5 %, *P* = 0.034) was higher in the elderly than in the non-elderly group (Table 1).

### Clinical outcomes

**Fig. 1:**
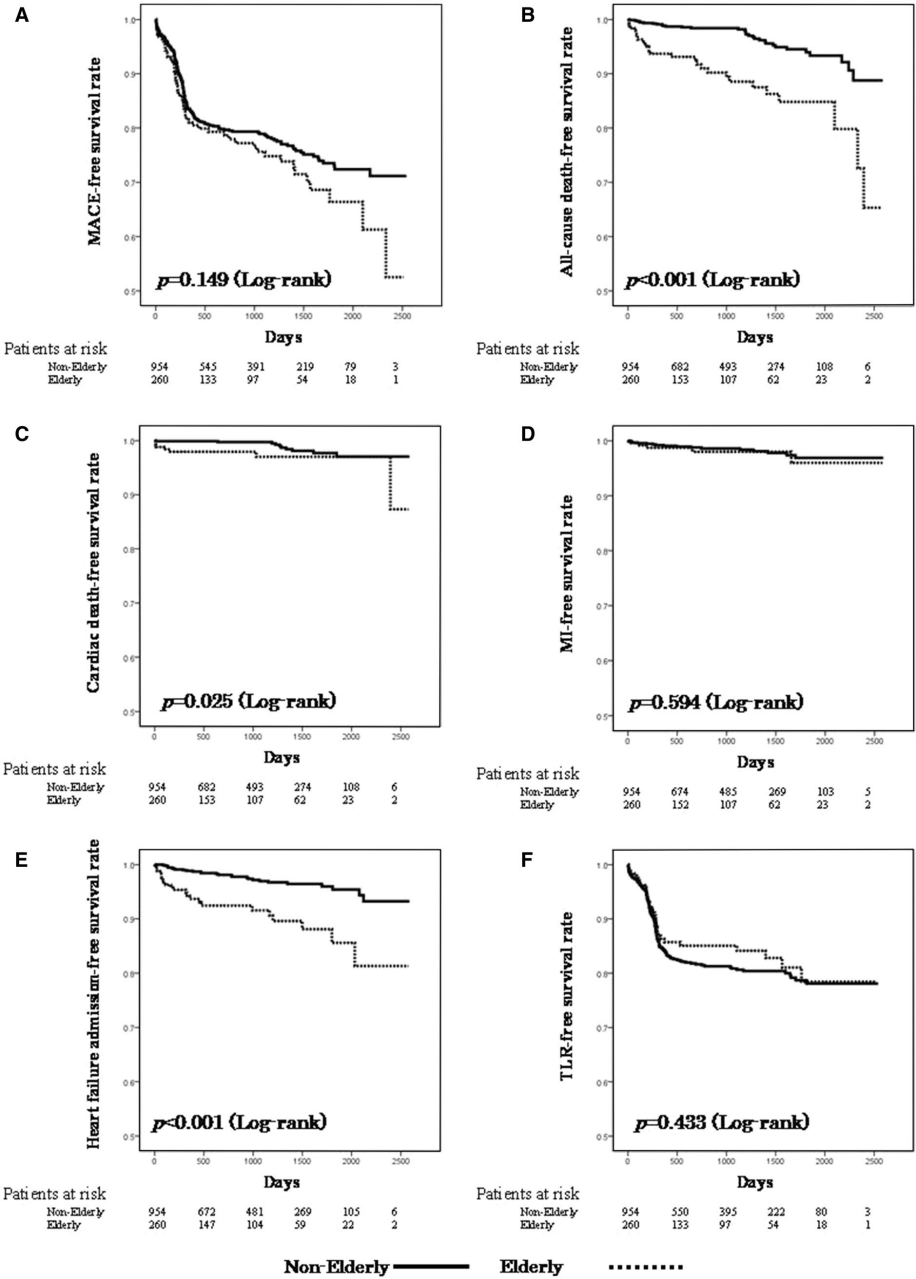
Kaplan–Meier curves for survival rates without major adverse cardiac events (*MACE*) (**a**), all-cause death (**b**), cardiac death (**c**), myocardial infarction (*MI*) (**d**), admission for heart failure (**e**), and target lesion revascularization (*TLR*) (**f**). *Solid line* elderly patients, *dotted line* non-elderly patients

Overall, all-cause death occurred in 28 elderly patients and 32 non-elderly patients. There were 60 and 194 composite end points, 7 and 10 cardiac deaths, 5 and 16 MI occurrences, 22 and 27 readmissions for heart failure, and 35 and 163 TLR occurrences in the elderly and non-elderly patients, respectively (Table 2). Kaplan–Meier curves and the log-rank test revealed that the frequency of MACE tended to be higher in the elderly than in the non-elderly patients (*P* = 0.149), and that the frequency of all-cause death (*P* < 0.001), cardiac death (*P* = 0.025), and readmission for heart failure (*P* < 0.001) were significantly higher in the elderly than in the non-elderly patients. Frequency of MI (*P* = 0.594) and TLR (*P* = 0.433) were comparable between the two groups (Fig. 1).

**Table 2:** Clinical outcomes

	Non-elderly (*n* = 954)	Elderly (*n* = 260)	*P* value
MACE	194/954 (20.3)	60/260 (23.1)	0.335
All-cause death	32/954 (3.4)	28/260 (10.8)	<0.001
Cardiac death	10/954 (1.0)	7/260 (2.7)	0.046
Cardiovascular death	12/954 (1.3)	7/260 (2.7)	0.099
MI	16/954 (1.7)	5/260 (1.9)	0.787
Readmission for heart failure	27/954 (2.8)	22/260 (8.5)	<0.001
TLR	163/954 (17.1)	35/260 (13.5)	0.161

Data are expressed as counts (percentage)

*MACE* major adverse cardiac event, *MI* myocardial infarction, *TLR* target lesion revascularization

### Predictors of all-cause death and cardiovascular death

Univariate Cox regression analysis showed that factors associated with all-cause death after PCI in the elderly patients included ACS, prior history of MI, eGFR, levels of total cholesterol and glucose, LVEF, and the use of statins, calcium-channel blockers, diuretics, and aldosterone antagonists (Table 3), whereas those in non-elderly patients included age, prior history of CABG surgery, dyslipidemia, cigarette smoking, eGFR, glucose level, LVEF, multivessel coronary disease, and the use of statins, β-blockers, and insulin (Table 5). Multivariate Cox regression analysis of the significant and marginally significant predictors (*P* < 0.10) in the univariate model revealed that the independent predictors of death in the elderly patients were prior history of MI, low eGFR, and poor LVEF in the elderly patients undergoing PCI (Table 4), whereas those in the non-elderly patients were prior history of PCI, higher glucose level, poor LVEF, and absence of statin treatment (Table 6). Cox regression analysis showed that independent predictors of cardiovascular death after PCI included poor LVEF in the elderly patients, and female gender, low eGFR, and poor LVEF in the non-elderly patients (see Supplementary table, Table S1-4).

**Table 3:** Unadjusted predictors for all-cause death of elderly patients

	*P* value	Hazard ratio	95 % CI
Age (years)	0.068	1.073	0.995–1.158
Male sex	0.505	0.772	0.360–1.654
Obesity	0.107	0.367	0.109–1.242
ACS	0.002	3.661	1.612–8.316
Prior MI	0.031	2.742	1.100–6.836
Prior PCI	0.882	0.896	0.211–3.806
Prior CABG	0.399	0.045	0.000–61.236
Hypertension	0.138	0.570	0.272–1.198
Diabetes mellitus	0.583	1.242	0.573–2.692
Dyslipidemia	0.066	0.484	0.223–1.050
Hyperuricemia	0.107	1.870	0.873–4.004
Cigarette smoking	0.661	0.789	0.274–2.275
Family history	0.768	0.835	0.252–2.769
eGFR (ml/min/1.73 m^2^)	0.005	0.972	0.953–0.991
Total cholesterol (mg/dl)	0.035	0.986	0.973–0.999
LDL cholesterol (mg/dl)	0.127	0.989	0.975–1.003
HDL cholesterol (mg/dl)	0.469	0.989	0.961–1.018
TG (mg/dl)	0.053	0.990	0.980–1.000
Glucose (mg/dl)	0.024	1.006	1.001–1.012
HbA1c (%)	0.742	0.936	0.634–1.384
LVEF (%)	<0.001	0.938	0.916–0.961
DAPT	0.370	0.613	0.211–1.787
Anticoagulant therapy	0.087	1.199	0.359–4.001
Statins	0.007	0.290	0.117–0.717
β-Blockers	0.177	0.554	0.235–1.307
ACE-Is	0.663	1.243	0.466–3.315
ARBs	0.514	0.767	0.346–1.700
RAS-Is	0.837	0.925	0.440–1.945
CCBs	0.013	0.315	0.127–0.781
Vasodilators	0.719	0.871	0.412–1.843
Diuretics	0.043	2.321	1.026–5.248
Aldosterone antagonist	0.001	4.267	1.778–10.238
Antidiabetic drugs	0.151	0.348	0.082–1.468
Thiazolidinediones	0.517	0.047	0.000–484.579
Insulin	0.899	0.875	0.113–6.783
LMT	0.057	2.434	0.975–6.075
MVD	0.728	1.157	0.508–2.635

*ACS* acute coronary syndrome, *Prior MI* prior history of myocardial infarction, *Prior PCI* prior history of percutaneous coronary intervention, *Prior CABG* prior history of coronary artery bypass graft, *eGFR* estimated glomerular filtration rate, *LDL* low-density lipoprotein, *HDL* high-density lipoprotein, *TG* triglyceride, *LVEF* left ventricular ejection fraction, *DAPT* dual antiplatelet therapy, *Statin* HMG-CoA inhibitor, *ACE-I* angiotensin-converting enzyme inhibitor, *ARB* angiotensin II receptor blocker, *RAS-I* renin–angiotensin system inhibitor, *CCB* calcium-channel blocker, *LMT* left main trunk disease, *MVD* multivessel disease, *CI* confidence interval

**Table 4:** Adjusted determinants of all-cause death of elderly patients

	Univariate *P* value	*P* value	Hazard ratio	95 % CI
Age	0.068	0.656	0.979	0.891–1.076
ACS	0.002	0.681	1.237	0.448–3.419
Prior MI	0.031	0.012	3.894	1.351–11.225
eGFR	0.005	0.010	0.967	0.944–0.993
Total cholesterol	0.035	0.955	1.000	0.984–1.017
TG	0.053	0.555	0.997	0.985–1.008
Glucose	0.024	0.759	1.001	0.994–1.008
LVEF	<0.001	<0.001	0.934	0.904–0.966
Anticoagulant therapy	0.087	0.970	0.970	0.202–4.6635
Statin	0.007	0.152	0.477	0.173–1.314
CCB	0.013	0.163	0.470	0.163–1.359
Diuretics	0.043	0.360	0.531	0.161–1.757
Aldosterone antagonist	0.001	0.576	1.430	0.408–5.017
LMT	0.057	0.338	0.418	0.109–1.598

*ACS* acute coronary syndrome, *Prior MI* history of myocardial infarction, *eGFR* estimated glomerular filtration rate, *TG* triglyceride, *LVEF* left ventricular ejection fraction, *DAPT* dual antiplatelet therapy, *Statin* HMG-CoA inhibitor, *CCB* calcium-channel blocker, *LMT* left main trunk disease, *CI* confidence interval

**Table 5:** Unadjusted predictors for all-cause death of non-elderly patients

	*P* value	Hazard ratio	95 % CI
Age	0.006	1.079	1.022–1.138
Male sex	0.878	0.928	0.357–2.413
Obesity	0.051	0.450	0.202–1.003
ACS	0.981	1.009	0.498–2.043
Prior MI	0.184	1.827	0.751–4.442
Prior PCI	0.098	2.115	0.870–5.139
Prior CABG	0.032	3.694	1.121–12.173
Hypertension	0.810	0.917	0.452–1.860
Diabetes mellitus	0.107	1.770	0.884–3.546
Dyslipidemia	0.037	0.472	0.233–0.956
Hyperuricemia	0.895	0.947	0.425–2.111
Cigarette smoking	0.010	0.333	0.144–0.771
Family history	0.053	0.140	0.019–1.025
eGFR	<0.001	0.967	0.955–0.980
Total cholesterol	0.281	0.995	0.985–1.004
LDL cholesterol	0.086	0.990	0.978–1.001
HDL cholesterol	0.237	1.012	0.992–1.031
TG	0.136	0.996	0.991–1.001
Glucose	<0.001	1.008	1.004–1.012
HbA1c	0.173	1.171	0.933–1.470
LVEF	<0.001	0.948	0.929–0.967
DAPT	0.828	0.853	0.203–3.579
Anticoagulant therapy	0.365	0.398	0.054–2.918
Statins	0.004	0.329	0.155–0.696
β-Blockers	0.009	2.611	1.275–5.345
ACE-Is	0.717	0.837	0.321–2.185
ARBs	0.529	0.780	0.360–1.689
RAS-Is	0.441	0.759	0.376–1.531
CCBs	0.856	0.937	0.462–1.898
Vasodilators	0.280	1.469	0.731–2.955
Diuretics	<0.001	5.318	2.563–11.038
Aldosterone antagonist	0.062	2.726	0.953–7.801
Antidiabetic drugs	0.374	1.438	0.646–3.202
Thiazolidinediones	0.174	2.286	0.694–7.534
Insulin	0.043	3.426	1.041–11.273
LMT	0.427	1.788	0.426–7.505
MVD	0.008	3.648	1.405–9.475

*ACS* acute coronary syndrome, *Prior MI* prior history of myocardial infarction, *Prior PCI* prior history of percutaneous coronary intervention, *Prior CABG* prior history of coronary artery bypass graft, *eGFR* estimated glomerular filtration rate, *LDL* low-density lipoprotein, *HDL* high-density lipoprotein, *TG* triglyceride, *LVEF* left ventricular ejection fraction, *DAPT* dual antiplatelet therapy, *Statin* HMG-CoA inhibitor, *ACE-I* angiotensin-converting enzyme inhibitor, *ARB* angiotensin II receptor blocker, *RAS-Is* renin–angiotensin system inhibitors, *CCB* calcium-channel blocker, *LMT* left main trunk disease, *MVD* multivessel disease, *CI* confidence interval

**Table 6:** Adjusted determinants of all-cause death of non-elderly patients

	Univariate *P* value	*P* value	Hazard ratio	95 % CI
Age	0.006	0.090	1.062	0.991–1.139
Obesity	0.051	0.144	0.485	0.183–1.281
Prior PCI	0.098	0.025	3.351	1.167–9.618
Prior CABG	0.032	0.195	0.296	0.047–1.868
Dyslipidemia	0.037	0.386	1.522	0.589–3.931
Cigarette smoking	0.010	0.379	0.649	0.248–1.700
Family history	0.053	0.955	0.000	
eGFR	<0.001	0.146	0.989	0.974–1.004
LDL	0.086	0.462	1.005	0.992–1.018
Glucose	<0.001	0.014	1.007	1.001–1.012
LVEF	<0.001	0.008	0.962	0.934–0.990
Statins	0.004	0.012	0.286	0.108–0.759
β-Blockers	0.009	0.080	2.114	0.915–4.882
Diuretics	<0.001	0.620	1.286	0.475–3.483
Aldosterone antagonist	0.062	0.146	0.285	0.052–1.550
Insulin	0.043	0.106	3.480	0.768–15.761
MVD	0.008	0.086	2.512	0.877–7.195

*Prior PCI* prior history of percutaneous coronary intervention, *Prior CABG* prior history of coronary artery bypass graft, *eGFR* estimated glomerular filtration rate, *LDL* low-density lipoprotein, *LVEF* left ventricular ejection fraction, *MVD* multivessel disease, *CI* confidence interval

## Discussion

The present study showed that elderly Japanese CAD patients who underwent PCI had higher incidences of all-cause death and cardiac death and higher rates of admission for heart failure than non-elderly patients. Multivariate Cox regression analysis showed that prior history of MI, low eGFR, and poor LVEF were independent predictors of all-cause death in elderly patients undergoing PCI.

Because the prevalence of CAD and other comorbidities is high among the elderly population, conservative pharmacologic treatment tends to be selected as the therapy of choice to reduce the risk of complications. However, because of advances in technical skills and better medical management, PCI has been associated with improved clinical outcomes in elderly patients [12, 13]. Moreover, elderly patients paradoxically had greater absolute risk reductions associated with surgical or percutaneous revascularization than younger patients in the setting of acute MI, owing to the higher baseline risk for significant morbidity and mortality [14, 15]. Because Japan has become an aging society with a consequent increase in the prevalence of CAD, the clinical significance of improved outcomes in elderly Japanese patients after PCI is very important.

In the present study, 21 % of the patients were older than 75 years, and elderly patients had higher incidences of all-cause death and cardiac death as well as higher rates of admission for heart failure than non-elderly patients. Although the incidences of all-cause death and cardiac death were higher in the elderly patients than in the non-elderly patients after PCI, the clinical outcomes were comparable with those of other clinical studies conducted in Western countries [14, 16]. Furthermore, considering that 41.5 % of elderly patients underwent PCI for ACS, this study suggests that the clinical outcomes of elderly Japanese patients after PCI were acceptable in clinical practice. Thus, revascularization therapy should be indicated in elderly patients when appropriate, and should not be avoided owing to higher age or greater morbidity.

Multivariate Cox regression analysis showed that independent predictors of death included prior history of MI, low eGFR, and poor LVEF in elderly patients, and prior history of PCI, higher glucose level, poor LVEF, and absence of statin treatment in non-elderly patients. Left ventricular function and renal function might have stronger predictive value in the elderly patients after PCI. Cardiac failure and renal dysfunction synergistically magnify the poor clinical outcomes associated with either disease alone [17].

Optimized medical therapy that includes the use of β-blockers, renin–angiotensin system inhibitor (RAS-I)s, and statins is crucial in elderly patients. The TIME trial strongly encouraged optimized medical therapy in elderly patients undergoing PCI [18]. As vasospastic angina is common in Japanese patients, administration of β-blockers tends to be avoided in clinical practice [19]. In fact, only 36.2 % of elderly patients used β-blockers, whereas 46.2 % of elderly patients used vasodilators. In addition, RAS-Is were administered to only 55.8 % of elderly CAD patients after PCI. Unfortunately, an increase in creatinine levels is sometimes associated with the administration of RAS-Is in the presence of underlying renal disease, thereby creating a therapeutic dilemma [20]. Because clinical studies support the benefits of β-blockers [21–23] and RAS-Is [24, 25] for CAD, the use of these agents is recommended in CAD patients when the side effects are acceptable, even in elderly patients and in those with chronic kidney disease (CKD). Statins are also promising agents for the treatment of CAD, and their effects have been studied extensively [26–28]. In Western countries, statins are administered to 80–90 % of CAD patients [29]. By contrast, only 50.4 % of the patients included in the present study received statins after PCI. Although statins were prescribed to patients, they were often not consumed or discontinued by the patients for various reasons. Caution should be exercised when prescribing statins to elderly patients with CKD because of the increased risk of toxicity, particularly the risk of myopathy. Optimized medical treatment is believed to reduce the morbidity and mortality of CAD patients, although only a limited number of patients included in the present study received the medications listed above. Thus there may be room for further improvement in drug therapy. Moreover, we do not have the data regarding the continued use of medications in this study. Further studies will be needed to clarify the impact of the optimal medical treatment on long-term clinical outcomes of Japanese elderly CAD patients after PCI.

In this study, we evaluated the clinical outcomes of elderly CAD patients undergoing PCI. However, Kimura et al. [30] reported better survival rates in patients older than 75 years undergoing CABG surgery, especially in high-risk patients, including those with triple-vessel disease and diabetes. Similarly, better survival after CABG in elderly individuals was also reported in the APPROACH registry [14] and the AWESOME trial [31]. Under clinical settings, elderly patients with significant comorbidities tend to be referred for PCI because of its less invasive nature. Such a selection bias may be responsible for the discrepancies between these studies. To justify the use of PCI as a treatment strategy, the clinical outcomes of elderly CAD patients treated using PCI and CABG should be compared.

### Study limitations

The present study has several limitations. The number of patients was small, so the statistical power may not be strong enough for any negative data to be conclusive. Further investigation of a large study population is necessary. In addition, our hospital is a single-department cardiovascular teaching facility, and the results of this study cannot be generalized to all medical centers.

## Conclusion

Elderly Japanese patients who underwent PCI had higher incidences of all-cause death and cardiac death in comparison with non-elderly patients. Poor left ventricular function and renal dysfunction were independent predictors of all-cause death in elderly patients, suggesting that left ventricular dysfunction and renal dysfunction may synergistically contribute to the adverse clinical outcomes in elderly patients undergoing PCI.

## Electronic supplementary material

Below is the link to the electronic supplementary material.

Supplementary material 1 (DOC 106 kb)
