# Supplementary material for: Impact of aging on the clinical outcomes of Japanese patients with coronary artery disease after percutaneous coronary intervention
Source: Heart Vessels. 2013 Apr 4;29(2):156–64. doi: 10.1007/s00380-013-0339-9 (PMC3948512; doi:10.1007/s00380-013-0339-9)
Supplement: Supplementary file 1 — Supplementary material 1 (DOC 106 kb) [file 380_2013_339_MOESM1_ESM.doc]

| **Supplementary Table 1. Unadjusted Predictors for Cardiovascular Death of Elderly Patients** | | | |
| --- | --- | --- | --- |
|  | *p* value | Hazard ratio | 95% CI |
| Age (years) | 0.753 | 0.969 | 0.799-1.176 |
| Male sex | 0.536 | 0.641 | 0.142-2.897 |
| Obesity | 0.228 | 2.733 | 0.532-14.037 |
| ACS | 0.128 | 3.577 | 0.694-18.447 |
| Prior MI | 0.085 | 4.443 | 0.813-24.291 |
| Prior PCI | 0.583 | 1.826 | 0.213-15.674 |
| Prior CABG | 0.698 | 0.046 | 0.000-273011.285 |
| Hypertension | 0.719 | 0.760 | 0.170-3.403 |
| Diabetes Mellitus | 0.479 | 1.718 | 0.385-7.679 |
| Dyslipidemia | 0.210 | 0.350 | 0.068-1.806 |
| Hyperuricemia | 0.347 | 2.062 | 0.456-9.326 |
| Cigarette smoking | 0.826 | 0.789 | 0.095-6.564 |
| Family history | 0.524 | 0.041 | 0.000-771.278 |
| eGFR (mL/min/1.73 m^2^) | 0.046 | 0.962 | 0.925-0.999 |
| Total cholesterol (mg/dL) | 0.004 | 0.955 | 0.925-0.985 |
| LDL cholesterol (mg/dL) | 0.027 | 0.965 | 0.935-0.996 |
| HDL cholesterol (mg/dL) | 0.255 | 0.966 | 0.909-1.026 |
| TG (mg/dL) | 0.127 | 0.983 | 0.962-1.005 |
| Glucose (mg/dL) | 0.001 | 1.014 | 1.006-1.023 |
| HbA1c (%) | 0.785 | 1.096 | 0.567-2.121 |
| LVEF (%) | <0.001 | 0.880 | 0.820-0.945 |
| DAPT | 0.583 | 24.273 | 0.000-2165495.9 |
| Anti-coagulant therapy | 0.601 | 0.042 | 0.000-5905.0 |
| Statins | 0.694 | 0.740 | 0.165-3.313 |
| Beta-blockers | 0.241 | 0.022 | 0.000-12.889 |
| ACE-Is | 0.491 | 0.038 | 0.000-423.8 |
| ARBs | 0.557 | 0.611 | 0.118-3.157 |
| RAS-I | 0.221 | 0.357 | 0.069-1.855 |
| CCBs | 0.864 | 0.877 | 0.194-3.959 |
| Vasodilators | 0.378 | 0.478 | 0.093-2.467 |
| Diuretics | 0.448 | 1.931 | 0.353-10.562 |
| Aldosterone antagonist | 0.622 | 0.043 | 0.000-11453.1 |
| Antidiabetic drugs | 0.448 | 0.037 | 0.000-184.281 |
| Thiazolidinediones | 0.767 | 0.047 | 0.000-28453544.0 |
| Insulin | 0.243 | 4.025 | 0.389-41.644 |
| LMT | 0.627 | 1.704 | 0.199-14.592 |
| MVD | 0.820 | 1.210 | 0.234-6.250 |
| ACS, acute coronary syndrome; Prior MI, prior history of myocardial infarction, Prior PCI, prior history of percutaneous coronary intervention, Prior CABG, prior history of coronary artery bypass graft; eGFR, estimated glomerular filtration rate; LDL; LDL, low-density lipoprotein; HDL, high-density lipoprotein; TG, triglyceride; LVEF, left ventricular ejection fraction; DAPT, dual antiplatelet therapy; Statin, HMG-CoA inhibitor; ACE-I, angiotensin-converting enzyme inhibitor; ARB, angiotensin II receptor blocker; RAS-I, renin–angiotensin system inhibitor; CCB, calcium channel blocker; LMT, left main trunk disease; MVD, multivessel disease.  CI, confidence interval. | | | |

| **Supplementary Table 2. Adjusted Determinants of Cardiovascular Death of Elderly Patients** | | | | |
| --- | --- | --- | --- | --- |
|  | Univariate *p* value | *p* value | Hazard ratio | 95% CI |
| Prior MI | 0.085 | 0.085 | 7.707 | 0.755-78.619 |
| eGFR (mL/min/1.73 m^2^) | 0.046 | 0.082 | 0.960 | 0.917-1.005 |
| Total cholesterol (mg/dL) | 0.004 | 0.323 | 0.982 | 0.946-1.018 |
| Glucose (mg/dL) | 0.001 | 0.053 | 1.012 | 1.000-1.024 |
| LVEF (%) | <0.001 | 0.014 | 0.909 | 0.842-0.981 |
| Prior MI, prior history of myocardial infarction, eGFR, estimated glomerular filtration rate; LVEF, left ventricular ejection fraction.  CI, confidence interval. | | | | |

| **Supplementary Table 3. Unadjusted Predictors for Cardiovascular Death of Non-Elderly Patients** | | | |
| --- | --- | --- | --- |
|  | *p* value | Hazard ratio | 95% CI |
| Age (years) | 0.416 | 1.032 | 0.957-1.113 |
| Male sex | 0.088 | 0.352 | 0.106-1.169 |
| Obesity | 0.089 | 0.267 | 0.059-1.220 |
| ACS | 0.977 | 1.017 | 0.323-3.204 |
| Prior MI | 0.160 | 2.552 | 0.691-9.429 |
| Prior PCI | 0.093 | 3.071 | 0.831-11.349 |
| Prior CABG | 0.009 | 7.718 | 1.682-35.416 |
| Hypertension | 0.712 | 1.254 | 0.377-4.168 |
| Diabetes Mellitus | 0.077 | 2.817 | 0.894-8.876 |
| Dyslipidemia | 0.962 | 0.973 | 0.309-3.066 |
| Hyperuricemia | 0.985 | 1.013 | 0.274-3.742 |
| Cigarette smoking | 0.052 | 0.221 | 0.049-1.011 |
| Family history | 0.277 | 0.035 | 0.000-14.797 |
| eGFR (mL/min/1.73 m^2^) | <0.001 | 0.944 | 0.925-0.963 |
| Total cholesterol (mg/dL) | 0.251 | 0.991 | 0.975-1.007 |
| LDL cholesterol (mg/dL) | 0.043 | 0.979 | 0.960-0.999 |
| HDL cholesterol (mg/dL) | 0.070 | 1.024 | 0.998-1.050 |
| TG (mg/dL) | 0.341 | 0.996 | 0.988-1.004 |
| Glucose (mg/dL) | <0.001 | 1.013 | 1.008-1.018 |
| HbA1c (%) | 0.032 | 1.391 | 1.029-1.881 |
| LVEF (%) | <0.001 | 0.934 | 0.906-0.964 |
| DAPT | 0.683 | 0.652 | 0.084-5.065 |
| Anti-coagulant therapy | 0.517 | 0.044 | 0.000-557.221 |
| Statins | 0.045 | 0.262 | 0.071-0.970 |
| Beta-blockers | 0.919 | 1.061 | 0.337-3.346 |
| ACE-Is | 0.697 | 1.297 | 0.350-4.799 |
| ARBs | 0.941 | 1.046 | 0.314-3.482 |
| RAS-I | 0.655 | 1.300 | 0.411-4.107 |
| CCBs | 0.550 | 1.412 | 0.455-4.381 |
| Vasodilators | 0.772 | 1.185 | 0.376-3.739 |
| Diuretics | 0.008 | 5.052 | 1.519-16.803 |
| Aldosterone antagonist | 0.119 | 3.356 | 0.733-15.367 |
| Antidiabetic drugs | 0.539 | 1.507 | 0.408-5.566 |
| Thiazolidinediones | 0.469 | 2.131 | 0.275-16.540 |
| Insulin | 0.015 | 6.624 | 1.451-30249 |
| LMT | 0.018 | 6.279 | 1.368-28.822 |
| MVD | 0.127 | 3.266 | 0.716-14.908 |
| ACS, acute coronary syndrome; Prior MI, prior history of myocardial infarction, Prior PCI, prior history of percutaneous coronary intervention, Prior CABG, prior history of coronary artery bypass graft; eGFR, estimated glomerular filtration rate; LDL; LDL, low-density lipoprotein; HDL, high-density lipoprotein; TG, triglyceride; LVEF, left ventricular ejection fraction; DAPT, dual antiplatelet therapy; Statin, HMG-CoA inhibitor; ACE-I, angiotensin-converting enzyme inhibitor; ARB, angiotensin II receptor blocker; RAS-I, renin–angiotensin system inhibitor; CCB, calcium channel blocker; LMT, left main trunk disease; MVD, multivessel disease.  CI, confidence interval. | | | |

| **Supplementary Table 4. Adjusted Determinants of Cardiovascular Death of Non-Elderly Patients** | | | | |
| --- | --- | --- | --- | --- |
|  | Univariate *p* value | *p* value | Hazard ratio | 95% CI |
| Male sex | 0.088 | 0.002 | 0.050 | 0.008-0.331 |
| Obesity | 0.089 | 0.245 | 0.276 | 0.032-2.414 |
| Prior PCI | 0.093 | 0.346 | 2.645 | 0.350-19.955 |
| Prior CABG | 0.009 | 0.766 | 0.540 | 0.009-31.424 |
| Cigarette smoking | 0.052 | 0.719 | 0.728 | 0.129-4.105 |
| eGFR (mL/min/1.73 m^2^) | <0.001 | 0.001 | 0.956 | 0.930-0.983 |
| LDL (mg/dL) | 0.043 | 0.504 | 0.992 | 0.970-1.015 |
| Glucose (mg/dL) | <0.001 | 0.005 | 1.012 | 1.004-1.021 |
| LVEF (%) | <0.001 | 0.0017 | 0.947 | 0.906-0.990 |
| Statins | 0.045 | 0.402 | 0.487 | 0.090-2.623 |
| Diuretics | 0.008 | 0.814 | 0.791 | 0.112-5.570 |
| LMT | 0.018 | 0.930 | 1.207 | 0.018-79.526 |
| Prior PCI, prior history of percutaneous coronary intervention, Prior CABG, prior history of coronary artery bypass graft; eGFR, estimated glomerular filtration rate; LDL; LDL, low-density lipoprotein; LVEF, left ventricular ejection fraction; LMT, left main trunk disease.  CI, confidence interval. | | | | |
